# Silent port catheter fracture with normal infusion: A case report and literature review

**DOI:** 10.1097/MD.0000000000045004

**Published:** 2025-10-03

**Authors:** Linlin Xiang, Haoping Chen, Jingjin Wu

**Affiliations:** a Department of Nursing, the Fourth Affiliated Hospital of School of Medicine, and International School of Medicine, International Institutes of Medicine, Zhejiang University, Yiwu, China; b Department of General Surgery, the Fourth Affiliated Hospital of School of Medicine, and International School of Medicine, International Institutes of Medicine, Zhejiang University, Yiwu, China.

**Keywords:** catheter fracture, endovascular intervention, silent, TIVAP

## Abstract

**Rationale:**

Although the totally implantable venous access ports have been widely used for chemotherapy and parenteral nutrition because of their safety and durability, some postoperative complications may still occur, such as catheter fracture, which can occur so silently and be discovered after several cycles of chemotherapy.

**Patient concerns:**

A 55-year-old male received the implantation of a chest wall venous port via the right internal jugular vein approach in an external hospital more than 3 years ago due to the need for chemotherapy for pancreatic cancer. On the 1221st catheter day, blood return could not be aspirated.

**Diagnoses:**

A chest X-ray confirmed the catheter rupture.

**Interventions:**

An emergency endovascular surgery was performed to remove the ruptured catheter and the port body, and a new venous port was implanted.

**Outcomes:**

Upon careful review of the medical history, the patient could not feel the cord-like structure in front of the right clavicle 3 months ago. Since the previous chemotherapy sessions had gone smoothly, the patient did not pay much attention to it. This also implies that the catheter rupture might have occurred at that time. Moreover, several chemotherapy infusions were still carried out after the catheter rupture, and the patient did not experience any discomfort.

**Lessons:**

The catheter rupture of venous ports can present silently, meaning the port can still be used for smooth infusion without any patient’s discomfort, which precisely delays the diagnosis, and may lead to life-threatening complications such as pulmonary embolism. Standardized maintenance of the venous port, regular chest imaging examinations, and careful interpretation of the imaging findings are helpful for the early diagnosis of such silent catheter ruptures.

## 1. Introduction

Since 1982, the totally implantable venous access ports (TIVAPs) have revolutionized the long-term venous access management for chemotherapy administration and total parenteral nutrition in oncology patients. This implantable device has demonstrated clinical reliability, operational safety, and extended service longevity, thereby substantially enhancing patient comfort and quality of life metrics in cancer care settings.^[1]^ Despite demonstrating a lower overall incidence of complications than the peripherally inserted central catheters, TIVAP-related adverse events persist within a clinically significant range of 1.8% to 14.4% in contemporary studies.^[2]^ Of particular clinical concern, catheter fracture represents a critical device failure event, with reported incidence rates ranging from 0.1% to 2.1% in contemporary case series.^[3,4]^ The incidence of catheter rupture was 1.8% in the internal jugular vein (IJV) and in the subclavian vein 1.1% to 5.0%.^[5]^ Although the rate is low, the catheter fracture can be fatal if the broken catheter migrates to the heart and causes arrhythmia or pulmonary embolism, respectively. Clinically, catheter integrity compromise manifests through loss of blood reflux, post-infusion infusate extravasation, port-site edema, and localized tenderness, which help in an early diagnosis of catheter fracture.

However, cases in which the time of catheter rupture is discovered a long time after it occurs are rarely reported. Herein, we will report such an uncommon but alarming case, in which therapeutic infusions were successfully maintained for months post-catheter fracture without treatment interruption.

## 2. Case presentation

A 55-year-old male presented with pancreatic adenocarcinoma confirmed by core needle biopsy 3 years ago. Then a TIVAP implantation (Bard Access Systems, Inc., Salt Lake City) via the IJV was successfully performed, followed by initiation of neoadjuvant chemotherapy (albumin-bound paclitaxel 200 mg/m^2^ + gemcitabine 1650 mg/m^2^) on day 1 and 8 of a 21-day cycle. Subsequently, the patient underwent pancreatoduodenectomy with portal vein reconstruction after 3 months, achieving uneventful postoperative recovery. Adjuvant chemotherapy (gemcitabine 1600 mg/m^2^ on days 1 and 8 + capecitabine 1500 mg/m^2^/day on days 1–14) was administered every 21 days in the following 6 months.

Six months ago, surveillance showed a sharp increase in CA19-9 levels, rising from 16.64 to 5744.7 U/mL. A subsequent positron emission tomography-computed tomography scan revealed soft tissue densities in the retroperitoneal region, indicating metastatic progression. Consequently, a modified AG regimen of gemcitabine (1600 mg/m^2^) and albumin-bound paclitaxel (200 mg/m^2^) was started on days 1 and 15 of a 28-day cycle.

**Figure 1.**
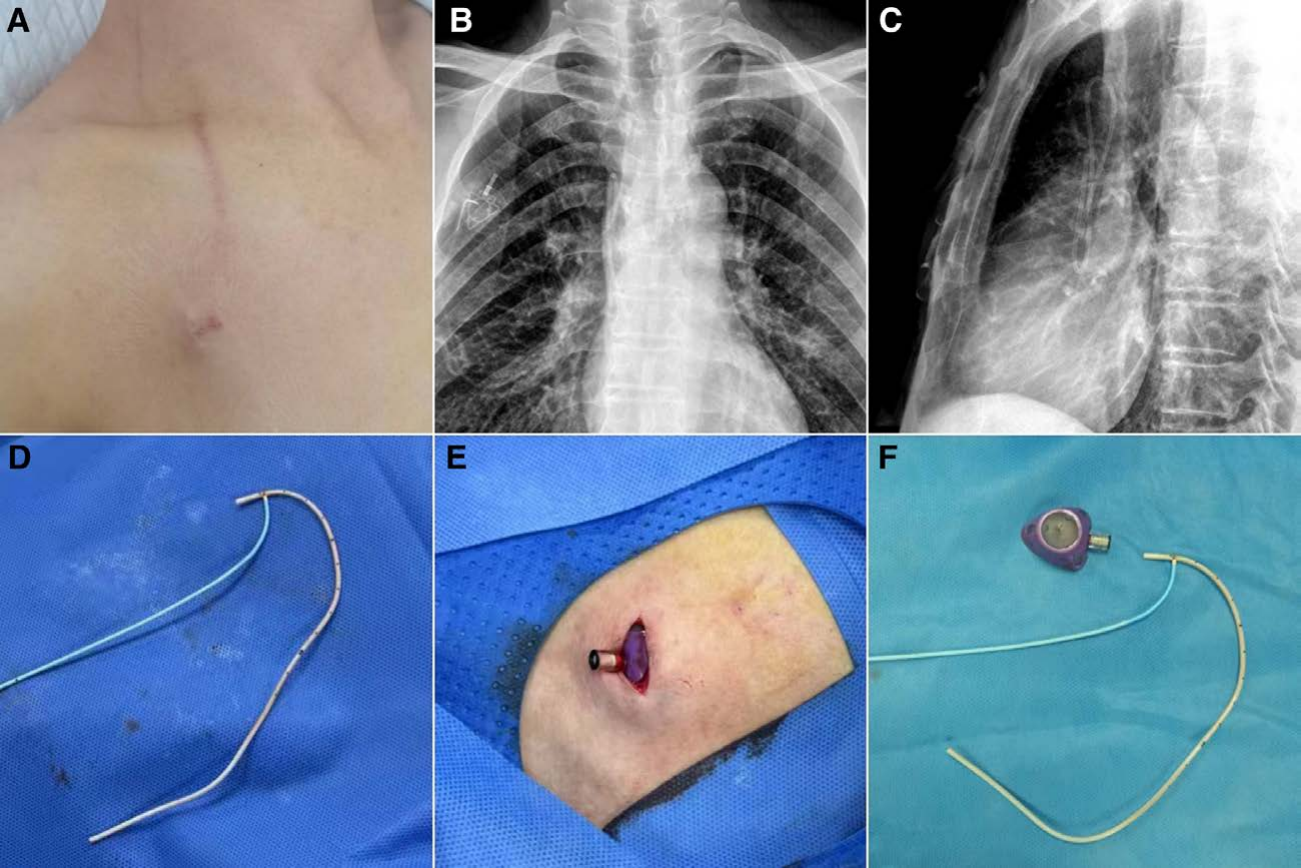
(A) The subcutaneous tunnel in front of the collarbone collapsed. (B) The frontal chest radiograph indicated that the catheter and the port body were disconnected. (C) The lateral chest radiograph showed that the broken catheter was approximately located in the SVC. (D) The catheter was removed by the snare device. (E) The isolated port body with a remaining part of the catheter; (F) The broken catheter and the isolated port body. SVC = superior vena cava.

On the 1221st catheter day, during routine catheter maintenance procedures, approximately 2 mL of dark-brown-colored blood was aspirated from the venous port. Clinical assessment revealed a concave deformation in the subcutaneous catheter tract region (Fig. 1A). Concurrently, visual inspection of the aspirated specimen revealed opalescent fluid with visible sedimentations. Following clinical notification of suspected port dysfunction, a chest radiograph was performed and a catheter fracture was observed (Fig. 1B) with the fractured catheter coiled within the superior vena cava (SVC) (Fig. 1C). The plan for the endovascular removal of the free ruptured catheter was promptly initiated 45 minutes after the diagnosis.

Following successful percutaneous access to the right common femoral vein, a guidewire-catheter coaxial technique was used to advance the catheter into the SVC for angiography. No peri-catheter thrombus was observed in the SVC (see Video S1, which demonstrates no catheter-associated venous thrombosis). The snare device was used for catheter extraction (see Video S2, which demonstrates an intravascular snare was used to capture one end of the catheter), and the retrieval of the free-floating catheter was ultimately accomplished through the established femoral access sheath (Fig. 1D) (see Video S3, which demonstrates the removal of the catheter from the sheath).

Utilizing the previous incision over the port pocket, the tissues were dissected in layers until the port reservoir was completely explanted. The catheter fractured at the luer lock connection (Fig. 1E) and the retrieved catheter tip demonstrated a 22.5-cm length matching original implantation records (Fig. 1F). The residual catheter tunnel orifice was systematically closed. Subsequently, the ultrasound-guided cannulation of the right axillary vein was performed, and a new venous port (ZS2 Series Implantable Drug Delivery Device; ZS2-II-1.8/1.2-750; Beijing Yue tong Medical Equipment Co., Ltd., Suzhou, Jiangsu, China) was implanted inside the original port pocket using the surgical technique method described previously.^[6]^ The total implanted catheter length was measured at 21.5 cm, with radiographic confirmation of the catheter tip positioning at the seventh thoracic vertebral body level. The incision was closed utilizing a subcuticular suture technique, followed by a subsequent standardized hemostatic compression applied at the right common femoral venous access site. During the approximately 4-month follow-up monitoring after the surgery, the patient demonstrated satisfactory wound epithelization and completed 4 chemotherapy cycles without any catheter-associated complications.

To investigate the causes of the condition, a retrospective analysis of the patient’s medical history was conducted. The patient reported that around 3 months ago, the subcutaneous catheter tract in the cervico-clavicular region suddenly disappeared, indicating a possible catheter rupture. However, this change was not reported to the clinical team due to the lack of symptoms like pain or dyspnea. Prior institutional records indicated that the venous port system was intact on CT on the 1107th catheter day (see Video S4, which demonstrates that the catheter and port body were well connected), but a fracture was noted by the 1193rd day (see Video S5, which demonstrates the disconnection of the catheter and port body). This critical finding was not documented in the clinical records. During the 16-week chemotherapy regimen, temporary catheter issues arose during routine port maintenance. However, after the nurse instructed the patient to move his neck and shoulder and cough, blood recovery improved significantly. Continuous cardiorespiratory monitoring confirmed the absence of acute distress, allowing for the seamless administration of cytotoxic therapy. This case exemplifies a striking and rare phenomenon: the successful delivery of chemotherapy through a fractured implantable venous port for 3 months.

## 3. Discussion and conclusions

Infusion port catheter fracture represents a rare late-phase complication. The interval between implantation and discovery of catheter fracture was 451.6 ± 325.4 days.^[7]^ To gain further insight into the clinical management of port catheter fracture, we reviewed a large number of literature between 1988 and 2024 and summarized their clinical characteristics (Tables 1 and 2). The most common fracture site of the catheter is the port catheter junction, with an incidence of about 83%.^[8]^ This observation aligns with our case study findings. A slight male predominance in adult cohorts was shown. Most patients with catheter ruptures showed mild symptoms, like chest or back pain, catheter dysfunction, and typically could not use the catheter afterward. Notably, 41% of patients exhibit no immediate symptoms, and the catheter fracture was identified by imaging examinations, which manifested as silence.

**Table 1:** The brief review of literature related to catheter fracture of implantable venous port (basic information).

No.	Year	Author	Article type	Number of case	Pocket position	Tunnel	Gender	Age (yr)	Manufacturer; specification
1	1988	Thomas et al	Case	1	Chest wall	N/A	M	24	N/A
2	1991	Dr Rene, Lafreniere	Case	1	N/A	N/A	F	35	Port-A-Cath; 2.8 mm
3	1992	Inoue et al	Case	1	Chest wall	Yes	M	45	Infuse-A-Port; 2.5 mm
4	1993	Röggla et al	Case	1	N/A	N/A	M	36	Port-a-Cath; N/A
5	1998	Nostdahl et al	Case	3	Chest wall	Yes	F, F, M	30, 69, and 42	Braun; N/A
6	1998	Vadlamani et al	Case	3	N/A	N/A	M, F, F	24, 31, and 41	Case 1: Pharmacia NuTech; Cases 2–3: N/A
7	1998	Biffi et al	Retrospective cohort study	5	N/A	N/A	N/A	N/A	Bard; 8 F
8	2000	Khanna et al	Case	1	Chest wall	N/A	M	70	Port-A-Cath; N/A
9	2003	Schummer et al	Case	2	Chest wall	N/A	F	61 and 40	N/A
10	2003	Bessoud et al	Retrospective cohort study	100	N/A	N/A	N/A	3 mo to 75 yr	N/A
11	2004	Filippou et al	Case	4	Chest wall	N/A	F	65, 68, 74, and 56	Port-A-Cath; N/A
12	2004	Yildizeli et al	Retrospective cohort study	4	Chest wall	Yes	N/A	N/A	N/A
13	2004	Gowda et al	Case	1	Yes	N/A	F	34	N/A
14	2005	Hackert et al	Case	1	Chest wall	No	F	49	Fresenius; N/A
15	2005	Kapadia et al	Case	1	Chest wall	Yes	F	16	HDC Corporation; N/A
16	2006	Kim et al	Case	1	Chest wall	N/A	F	39	Braun; 8.5 F
17	2006	Dillon et al	Retrospective cohort study	4	Chest wall	N/A	N/A	Average 11.8	Bard; 6.6 F
18	2006	Surov et al	Retrospective cohort study	11	Chest wall	N/A	F: 3, M: 8	Average 53	Fresenius Kabi GmbH, Baxter, Germany, pfm AG; N/A
19	2009	Seck et al	Case	3	Forearm,	N/A	F	84: case 2; N/A: cases 1 and 3	N/A
20	2009	Cheng et al	Retrospective cohort study	92	N/A	N/A	M: 47, F: 45	53.8 ± 13.5	Bard, Arrow International, Deltec; N/A
21	2010	Karaman et al	Retrospective cohort study	2	Chest wall	Yes	N/A	N/A	Braun; N/A
22	2011	Subramaniam et al	Retrospective cohort study	9	Chest wall	N/A	F	N/A	Bard; 7 F or 8 F
23	2011	Wu et al	Retrospective cohort study	59	N/A	N/A	M: 21; F: 38	54.32 ± 13.11	Arrow International Fr. 8.1: 51 cases, Bard Fr. 8: 5 cases, Bard Fr. 6.6: 3 cases
24	2012	Busch et al	Retrospective cohort study	6	Uparm	Yes	M: 5 cases; N/A: 1 case	70 and 57: 2 cases; N/A: 4 cases	Cook; 5.0/6.5 F
25	2012	Schenck et al	Retrospective cohort study	1	Chest wall	No	N/A	N/A	FA PHS Medical; N/A
26	2012	Kim et al	Retrospective cohort study	1	Chest wall	N/A	N/A	N/A	N/A
27	2013	Burbridge et al	Retrospective cohort study	11	Arm	Yes	M: 2, F: 9	Average 54.5	Cook Canada; 5 F
28	2014	Pignataro et al	Case	1	Chest wall	N/A	M	41	Bard; 8 F
29	2014	Balsorano et al	Retrospective cohort study	12	Chest wall	N/A	F: 7; M: 5	59.3 ± 11.1	Bard; N/A
30	2014	Nagasawa et al	Retrospective cohort study	4	N/A	N/A	N/A	N/A	BMedicon; N/A
31	2015	Tazzioli et al	Case	1	Chest wall	N/A	F	50	Bard; 8 F
32	2015	Ghaderian et al	Case	1	N/A	N/A	F	8	N/A
33	2016	Ko et al	Case	1	Chest wall	Yes	F	50	DistricAth; 9 F
34	2016	Kojima et al	Retrospective cohort study	16	Chest wall	Yes	N/A	N/A	Bard; 8 F
35	2016	Mery et al	Case	1	Chest wall	N/A	F	52	N/A
36	2017	Fujimoto et al	Case	1	Chest wall	N/A	F	61	N/A
37	2018	Barton et al	Retrospective cohort study	1	N/A	N/A	M	N/A	N/A
38	2018	Garcez et al	Case	1	Chest wall	N/A	F	57	N/A
39	2018	Wu et al	Retrospective cohort study	17	N/A	N/A	M: 13 cases, F: 4 cases	Average 48	N/A
40	2019	Lukito et al	Case	1	N/A	N/A	F	33	N/A
41	2019	Sun et al	Retrospective cohort study	1	Chest wall	Yes	N/A	N/A	Bard; 6 F
42	2019	Saijo et al	Case	3	Chest wall	Yes	M, F, F	64, 78, and 59	Bard; N/A
43	2021	Chuah et al	Case	1	Chest wall	Yes	M	50	N/A
44	2021	Sudhakar et al	Case	1	Chest wall	N/A	F	60	N/A
45	2021	Chen et al	Case	1	Chest wall	N/A	F	43	N/A
46	2022	Shah et al	Case	1	N/A	N/A	F	51	N/A
47	2022	Azeemuddin et al	Case	1	Chest wall	N/A	F	67	N/A
48	2022	Li et al	Retrospective cohort study	31	Chest wall	Yes	N/A	N/A	N/A
49	2022	Goyal et al	Case	1	N/A	N/A	F	3	N/A
50	2023	Takahashi et al	Case	1	Chest wall	Yes	M	83	Bard; 8.0 F
51	2023	Abbasov et al	Case	1	Chest wall	N/A	F	53	N/A
52	2023	Matta et al	Case	1	Chest wall	N/A	M	85	N/A
53	2024	Kordykiewicz et al	Case	1	Chest wall	Yes	M	67	Braun; N/A
54	2024	Dave et al	Case	1	Chest wall	Yes	F	56	N/A

F = female, M = male, N/A = not mentioned in literature.

**Table 2:** The brief review of literature related to catheter fracture of implantable venous port (catheter fracture information).

No.	Year	Author	Venous approach	Time of fracture discovery	Position of the free catheter	Fracture site	Patient symptoms at the time of rupture	Continous infusion through the port	Reasons for catheter breakage	Treatment for broken catheter	Treatment for portal
1	1988	Thomas et al	SCV	13 mo	Right atrium, right ventricle	Between the first rib and collarbone	Pain; no blood return	No	Shear force resulting from movement of the shoulder and pectoralis major	Endovascular removal	Surgical removal
2	1991	Dr Rene, Lafreniere	Right SCV	11 mo	Right atrium	Between the first rib and collarbone	Infusion pain	No	Pinch-off syndrome	Endovascular removal	N/A
3	1992	Inoue et al	Left SCV	19 mo	Just catheter crack	Between the first rib and collarbone	Pain, dysfunction	No	Pinch-off syndrome	Surgical removal	Surgical removal
4	1993	Röggla et al	Left SCV	12 mo	Pulmonary artery	Venous entrance	Chest X-rays	N/A	Pinch-off syndrome	Endovascular removal	Surgical removal
5	1998	Nostdahl et al	SCV	5–6 wk, 7 mo, 1 mo	Just catheter crack	Between the first rib and collarbone	Tenderness, Pain during infusion, Difficulty in drawing blood; Low infusion speed, Redness of the skin;	No	Pinch-off syndrome	Surgical removal	Surgical removal
6	1998	Vadlamani et al	N/A	Case 1: 4 mo; Case 2: N/A; Case 3: 4 wk	Case 1: Pulmonary artery; Case 2: Right atrium; Case 3: N/A	N/A	Case 1: No return of blood; Case 2: Chest X-rays; Case 3: Dysfunction	No	N/A	Cases 1–2: Endovascular removal	N/A
7	1998	Biffi et al	SCV, Cephalic vein	66 ± 18 d in 2 cases; 10 mo in another case;	N/A	N/A	Palpitations and Chest discomfort in 2 cases; Asymptomatic in the other 3 cases	N/A	Pinch-off syndrome for 1 case	Endovascular removal	N/A
8	2000	Khanna et al	Right SCV	31 wk	Right atrium	Between the first rib and collarbone	Right shoulder pain	No	Pinch-off syndrome	Endovascular removal	N/A
9	2003	Schummer et al	Right SCV	1 wk after chemotherapy ends; 12 mo	Right ventricle; left pulmonary artery	Middle segment	Difficult injection	No	N/A	Endovascular removal	Surgical removal
10	2003	Bessoud et al	SCV 、IJV	290 ± 200 d	pulmonary artery: 26, right ventricle: 24, right atrium: 23, IVC: 7, SVC: 17, hepatic vein: 2, thymic vein: 1	N/A	Resistance in the injection and No blood return.	No	Accidental sectioning during retrieval: 7, Loss during implantation: 3, N/A-90	Endovascular removal: 95, not retrieved: 5.	N/A
11	2004	Filippou et al	SCV	6, 18, 14, 8, and mo	1. Right atrium 2. Right ventricle 3. Right atrium 4. Partial segment remained in subcutaneous tissue	N/A	Back pain: 1, Infusion resistance: 3	No	Reduced elasticity of the catheter and Wrong manipulations	Thoracotomy: 1-failed and receiving anticoagulant therapy; Endovascular removal: 3	Surgical removal
12	2004	Yildizeli et al	N/A	126, 140, 154, and 175 d	3 in SVC and 1 in pulmonary artery	N/A	Asymptomatic, Impaired return of blood and detected by a routine chest X-ray	No	Pinch-off syndrome	Endovascular removal	N/A
13	2004	Gowda et al	Right SCV	3 yr	Right ventricle	Between the first rib and collarbone	Shortness of breath, palpitations, ventricular tachycardia	No	Pinch-off syndrome	Endovascular removal	Surgical removal
14	2005	Hackert et al	Cephalic vein	2 yr	Right upper lobe of the lung	N/A	Intermittent coughing, Shortness of breath, General weakness, Failing blood aspiration	No	N/A	Thoracotomy	Remain
15	2005	Kapadia et al	Right SCV	24 mo	Istal catheter embedded into the myocardium of the right ventricle, whereas the proximal end was stuck to the wall of the SVC.	Catheter to port connection	Asymptomatic	No	Irregular maintenance	Endovascular removal failed, and it was removed by thoracotomy	Surgical removal
16	2006	Kim et al	SCV	3 mo	Pulmonary artery	Between the first rib and collarbone	Chest and chin pain	No	Pinch-off syndrome	Endovascular removal	N/A
17	2006	Dillon et al	N/A	Average 1075 d	Pulmonary artery or heart	Catheter to port connection: 3 cases, proximal to the clavicle: 1 case	Pain or Resistance to Infusion	No	Excessive pressure at the connection between the catheter and the port, conventional wear	Endovascular removal	N/A
18	2006	Surov et al	SCV	Average 203 d	Pulmonary artery	Between the first rib and collarbone in 9 cases; Catheter to port connection in 2 cases	Catheter dysfunction in 7 cases; asymptomatic in 4 cases	No	Pinch-off syndrome iin 9 cases; incorrect locking of the steel ring or flaws in the system itself in 2 cases	Endovascular removal	N/A
19	2009	Seck et al	Vena brachialis-case 2; N/A-cases 1 and 3	N/A	Pulmonary artery: cases 1 and 2; dislocation of the connection: case 3	1. Proximal catheter; 2. N/A; 3. Catheter to port connection	Infusion resistance; Weakness, Dizziness, Breathing difficulties, No return of blood	No	Problems with the material.	N/A	Surgical removal
20	2009	Cheng et al	SCV	451.6 ± 325.4	Right atrium, IVC, SVC	Catheter to port connection: 77 cases; proximal part: 12 cases; Distal portion: 3 cases	Resistance during infusion: 51; Asymptomatic: 33; The rest unspecified	No	Faulty connection and alignment; Unskillful operator, forceful port irrigation and lower rate of cut-down approach	Endovascular removal in 90 cases; endovascular removal failed in 2 cases;	N/A
21	2010	Karaman et al	IJV	Second and third day	Just catheter crack	Venous entrance	Swelling and pain in the neck	No	Forceful saline infusion	Surgical removal	Surgical removal
22	2011	Subramaniam et al	SCV	Average 197 d	Right ventricle: 4 cases, Pulmonary artery: 3 cases	N/A	Asymptomatic-most	No	The design and manufacturing deficiencies of this catheter	Endovascular removal	N/A
23	2011	Wu et al	Right SCV: 26 cases, left SCV: 3 cases, right IJV: 1 case, right cephalic vein: 22 cases, left cephalic vein: 7 cases	496.03 ± 321.41	No dislodged catheter: 7 cases, Right atrium: 19 cases, IVC: 20 cases, Right ventricle: 12 cases, Pulmonary artery: 1 case	Lock nut area: 52 cases; Proximal end of catheter: 3 cases, Perforation only: 4 cases	Catheter dysfunction: 9; Asymptomatic: 49; Infection: 1 case	No	Port type Arrow Fr. 8.1, female gender, and implantation of the intravenous port via the subclavian route	Endovascular removal: 58 cases; catheter shortening: 1 case	N/A
24	2012	Busch et al	Brachial vein	N/A	Pulmonary artery: 2 cases; Just catheter crack: 4 cases	N/A: 2 cases, Proximal leakage: 4 cases	Pain, Swelling and Contrast agent extravasation: 4; Chest X-rays: 2	No	Excessive shoulder movement: 2; N/A: 4	Endovascular removal	Surgical removal
25	2012	Schenck et al	Cephalic vein	N/A	Pulmonary artery	N/A	N/A	N/A	N/A	N/A	N/A
26	2012	Kim et al	SCV	6 mo	Right atrium	Between the first rib and collarbone	Palpitations and Chest tightness	No	Pinch-off syndrome	Endovascular removal	N/A
27	2013	Burbridge et al	N/A	Average 682 d	3 in peripheral venous system; 8 in pulmonary artery	Venous entrance	Chest X-rays	N/A	Arm movement or narrow and fibrotic veins	Endovascular removal	N/A
28	2014	Pignataro et al	Right IJV	24 mo	Right atrium, within the coronary sinus	Middle segment	Neck pain and Swelling during infusion	No	N/A	Endovascular removal	N/A
29	2014	Balsorano et al	IJV	589–1842 d	N/A	N/A	Catheter blockage: 3; Drug extravasation: 2; Asymptomatic: 7	N/A: 7; No: 5	“Out-of-plane” approach and type of port	N/A	N/A
30	2014	Nagasawa et al	IJV: 3 cases; SCV: 1 case	N/A	N/A	N/A	N/A	N/A	Chronic stress in IJV approach; Pinch-off syndrome in SCV approach	N/A	N/A
31	2015	Tazzioli et al	Right IJV	35 mo	Right ventricle	The proximal third	Catheter dysfunction	No	N/A	Endovascular removal failed and follow-up	N/A
32	2015	Ghaderian et al	N/A	3 yr	Right atrium	N/A	Asymptomatic	No	N/A	Endovascular removal	Surgical removal
33	2016	Ko et al	Right IJV	3 mo after final chemotherapy	Right atrium	Catheter to port connection	Swelling of the right neck	No	Irregular maintenance	Endovascular removal	Surgical removal
34	2016	Kojima et al	Right IJV	Average 621 d	Right chamber of the heart, pulmonary artery, or coronary sinus	Venous entrance: 14 cases; Proximal catheter: 2 cases	Subcutaneous swelling, Skin reddening and/or Pain at the port or along the subcutaneous Catheter tract: 12 cases; Asymptomatic: 4 cases	No	Weaknesses of catheter material, pressure-sensitive valve design, bending stress concentration at the venous entrance, excessive depth at the catheter tip position, low body mass index, age and mobility, indwelling time.	Endovascular removal: 8 cases, left the catheter in coronary sinus: 1 case	Surgical removal
35	2016	Mery et al	Left SCV	9 mo	The great saphenous vein on the medial side of the right knee	Between the first rib and collarbone	Coughing, Infusion resistance	No	Pinch-off syndrome	Surgical removal	Surgical removal
36	2017	Fujimoto et al	Right SCV	63 mo	Pulmonary artery	Proximal catheter	Infusion resistance	No	Thrombotic occlusion	Endovascular removal	Surgical removal
37	2018	Barton et al	N/A	26 mo	N/A	N/A	N/A	N/A	N/A	N/A	Surgical removal
38	2018	Garcez et al	SCV	26 mo	IVC	Between the first rib and collarbone	Asymptomatic	N/A	Pinch-off syndrome	Endovascular removal	Surgical removal
39	2018	Wu et al	SCV	2 h to 53 mo	SVC, IVC, right atrial brachial vein, right ventricle, pulmonary artery	Middle segment, Distal 2/3: 13 cases	Asymptomatic-Most, Chest discomfort or Palpitations-a few	N/A	Pinch-off syndrome: 13 cases, the rest unspecified.	Endovascular removal	Surgical removal
40	2019	Lukito et al	N/A	1 yr	Coronary sinus	N/A	Asymptomatic	No	N/A	Endovascular removal failed and the patient refused thoracotomy	Surgical removal
41	2019	Sun et al	Right IJV	N/A	N/A	N/A	N/A	N/A	N/A	N/A	N/A
42	2019	Saijo et al	IJV	21, 31, and 38 mo	Right atrium, IVC, right ventricle	Middle segment	Chest X-ray in case 1, Catheter dysfunction in cases 2–3	No	1. Long-term repeated stretching force 2. Groshong silicone tube material is fragile	Endovascular removal	Surgical removal
43	2021	Chuah et al	SCV	4 yr	Right ventricle	Between the first rib and collarbone	Occasional palpitations and chest discomfort	No	Pinch-off syndrome	Endovascular removal	Surgical removal
44	2021	Sudhakar et al	SCV	14 mo	Coronary sinus, right ventricle	Between the first rib and collarbone	Asymptomatic	N/A	Pinch-off syndrome	Endovascular removal	Surgical removal
45	2021	Chen et al	Right IJV	163 d	Pulmonary artery	N/A	Wheezing, Coughing, Chest tightness, Shortness of breath, Difficulty breathing after exercise	No	N/A	Endovascular removal	N/A
46	2022	Shah et al	N/A	N/A	Right atrium, Right ventricle	Middle segment	Infusion resistance	No	N/A	Endovascular removal	N/A
47	2022	Azeemuddin et al	N/A	24 mo	Right atrium	Proximal catheter	No blood return; drowsiness; Loss of appetite	No	N/A	Endovascular removal	Surgical removal
48	2022	Li et al	SCV	N/A	N/A	Proximal end of the catheter: 17 cases; Between the first rib and collarbone: 14 cases	N/A	N/A	Clamping syndrome: 14; Separation of infusion port from the proximal catheter: 17	Endovascular removal	Surgical removal
49	2022	Goyal et al	N/A	25 d	Right ventricle, pulmonary artery	N/A	Severe chest pain and fever	No	N/A	Thoracotomy	N/A
50	2023	Takahashi et al	Right IJV	96 mo	Coronary sinus	Middle segment	Ventricular fibrillation	No	N/A	Thoracotomy	Surgical removal
51	2023	Abbasov et al	Right SCV	36 mo	Pulmonary artery	N/A	N/A	No	Pinch-off syndrome	Remain	Surgical removal
52	2023	Matta et al	Right SCV	36 d	Right ventricle	Between the first rib and collarbone	No blood return	No	Pinch-off syndrome	Endovascular removal	Surgical removal
53	2024	Kordykiewicz et al	N/A	1 mo	Pulmonary artery	N/A	Persistent cough, Weakness, Nausea and Heartburn	No	N/A	Thoracotomy	Surgical removal
54	2024	Dave et al	Right IJV	N/A	Right ventricle, pulmonary artery	N/A	Catheter dysfunction	No	N/A	Endovascular removal	N/A

IJV = internal jugular vein, IVC = inferior vena cava, N/A = not mentioned in literature, SCV = subclavian vein, SVC = superior vena cava.

This case is unique as the infusion port catheter was fractured, yet normal infusions continued for 3 months. Unlike most reports where issues arise immediately after fracture, this suggests that assessing catheter rupture should not rely solely on symptoms or routine exams but also consider individual circumstances. This case offers some new perspectives on catheter rupture management in infusion ports, enhancing our understanding and aiding in the improvement of clinical strategies.

Fracture of an implantable port catheter may have several causes, except for the pinch-off syndrome associated with the subclavian vein approach. Previous studies have demonstrated that catheter breakage is associated with multiple factors. On one hand, it correlates with patients’ occupation, low body mass index, age, lifestyle habits, and mobility, as well as prolonged indwelling time. On the other hand, excessive depth of the catheter tip position, material weaknesses, design and manufacturing deficiencies of the catheter, and bending stress concentration at the venous entrance also contribute to catheter failure. However, in recent years, advancements in material science, technological improvements, and enhanced management practices have significantly reduced the incidence of catheter breakage in infusion ports. This patient’s job involved repetitive arm movements for 4 months before the catheter rupture, likely contributing to mechanical stress and material fatigue in the catheter wall, which may lead to its failure. Flushing a catheter with a small syringe can create excessive pressure, weakening it and increasing the risk of breakage. Additionally, the materials used and repeated tensile stress can further deteriorate the catheter’s strength, leading to fractures.^[9–11]^ A catheter fracture at the port catheter junction can result from external compression and material fatigue due to repeated bending from shoulder movement. The port implantation method and the distance from the clavicle also increase the risk of fracture, as sharp angles can create higher local pressure, leading to fatigue cracks.^[12]^ In the retrospective study by Matsunari et al, venipuncture performed within 3 cm of the clavicle combined with maintaining a gentle catheter curvature (approximately 90°) was associated with reduced catheter fracture risk.^[13]^

In this patient, chemotherapy administration over 3 months occurred without adverse reactions, such as drug extravasation or phlebitis, despite a catheter fracture. This unusual situation may be due to an inflammatory response, fibroblasts, and granulation tissue around the catheter, creating a false passage between the port and the IJV. When poor blood aspiration occurs, changing body position or coughing can increase thoracic pressure, pumping blood from the IJV into the port so that blood can still be aspirated. The fibrotic tunnel wall may prevent drug penetration and diffusion, allowing the patient to avoid significant pain or discomfort. From another bold perspective, it is also worth considering whether, by simply removing the fractured catheter, the port can be continuously used for infusion until the end of chemotherapy. Of course, this requires verification with a large amount of data and ethical considerations.

The patient continued chemotherapy despite a catheter fracture but did not develop complications like phlebitis or pulmonary embolism. This case is interesting, and the result is fortunate for the patient, yet alarming for doctors. It offers valuable lessons and suggests areas for improvement in medical practice. The 2024 Infusion Nurses Society guidelines recommend an annual chest X-ray assessment of port position and integrity. If blood return is poor during port use, have the patient change positions (e.g., raise their arm or take deep breaths).^[12]^ This alters thoracic pressure or vessel shape, helping the catheter tip disengage from the vessel wall. Reviewing prior port maintenance, nurses may have performed improper blood aspiration. Before infusion, discard 2 to 3 mL of venous blood. However, nurses may not always collect enough to identify port issues. If there is poor blood return from the TIVAP, an X-ray should be considered to check for catheter fractures.^[14]^ Patient education after venous port implantation is vital. It should cover port types, complication recognition, and daily activity precautions. Strong cannulation skills and prompt medical treatment can help prevent port catheter fractures and serious complications.^[15]^

To summarize, catheter fracture of TIVAPs may manifest as clinically silent device failure without overt perivascular chemotherapeutic extravasation. This asymptomatic catheter disruption underscores the critical necessity for full patient education, standardized maintenance of the infusion port, and timely imaging examination, which are crucial. They help detect and intervene early, thus preventing serious complications.

## Author contributions

**Conceptualization:** Linlin Xiang, Jingjin Wu.

**Data curation:** Haoping Chen, Linlin Xiang.

**Methodology:** Jingjin Wu.

**Supervision:** Jingjin Wu.

**Validation:** Jingjin Wu.

**Visualization:** Haoping Chen, Linlin Xiang.

**Writing – original draft:** Haoping Chen, Linlin Xiang.

**Writing – review & editing:** Jingjin Wu.
